# Analysis of the real number of infected people by COVID-19: A system dynamics approach

**DOI:** 10.1371/journal.pone.0245728

**Published:** 2021-03-18

**Authors:** Bo Hu, Matthias Dehmer, Frank Emmert-Streib, Bo Zhang

**Affiliations:** 1 Department of Business Administration, Universität der Bundeswehr München, Neubiberg, Germany; 2 Department of Computer Science, Swiss Distance University of Applied Sciences, Brig, Switzerland; 3 School of Science, Xian Technological University, Xian, Shaanxi, China; 4 College of Artificial Intelligence, Nankai University, Tianjin, China; 5 Department of Biomedical Computer Science and Mechatronics, The Health and Life Science University, UMIT, Hall in Tyrol, Austria; 6 Predictive Medicine and Data Analytics Lab, Department of Signal Processing, Tampere University, Tampere, Finland; 7 Institute of Biosciences and Medical Technology, Tampere, Finland; 8 Information Center, Ministry of Ecology and Environment, Beijing, China; "INSERM", FRANCE

## Abstract

At the beginning of 2020, the COVID-19 pandemic was able to spread quickly in Wuhan and in the province of Hubei due to a lack of experience with this novel virus. Additionally, authories had no proven experience with applying insufficient medical, communication and crisis management tools. For a considerable period of time, the actual number of people infected was unknown. There were great uncertainties regarding the dynamics and spread of the Covid-19 virus infection. In this paper, we develop a system dynamics model for the three connected regions (Wuhan, Hubei excl. Wuhan, China excl. Hubei) to understand the infection and spread dynamics of the virus and provide a more accurate estimate of the number of infected people in Wuhan and discuss the necessity and effectivity of protective measures against this epidemic, such as the quarantines imposed throughout China. We use the statistics of confirmed cases of China excl. Hubei. Also the daily data on travel activity within China was utilized, in order to determine the actual numerical development of the infected people in Wuhan City and Hubei Province. We used a multivariate Monte Carlo optimization to parameterize the model to match the official statistics. In particular, we used the model to calculate the infections, which had already broken out, but were not diagnosed for various reasons.

## Introduction

At the beginning of 2020, the COVID-19 pandemic was able to spread quickly in Wuhan and in the province of Hubei due to a lack of experience with this novel virus. For quite a bit of time, the current number of people infected was unknown. In fact, e.g., authorities in China found severe uncertainties regarding the dynamics and spread of the SARS-CoV-2 virus which causes COVID-19. In this paper, we give a more accurate estimate of the number of infected people in Wuhan and discuss the effects and the need of protective measures against this epidemic, e.g., such as the quarantines imposed by the Chinese government.

Several important studies about COVID-19 have already been published. Wu et al. [1] used the SEID metapopulation model applied to international travel data from Wuhuan and estimated that the basic reproductive number for COVID-19 was 2.68 (95% CrI 2.47-2.86); that means 75 815 individuals (95% CrI 37 304—130 330) were infected in Wuhan as of January 25th, 2020, see [1]. Li et al. [2] used observations of reported infections in China based on Chinese mobility data. By utilizing a network-based, dynamical metapopulation model using Bayesian inference, they obtained that 86% of all infections were simply not documented (95% CI: [82%-90%]) before the travel restrictions in Wuhan were arranged by January 23rd, 2020, see [2]. Lai et al. [3] investigated the temporal original rate of viral evolution and population dynamics of SARS-CoV-2 using 52 full genomes of viral strains sampled in different countries, and estimated the R value equal to 2.6 (range, 2.1-5.1) [3]. Qun Li et al. collected information on demographic characteristics, exposure history, and illness timelines of the first 425 laboratory-confirmed COVID-19 cases in China and estimated that the mean incubation period was 5.2 days (95% confidence interval [CI], 4.1 to 7.0) and the basic reproductive number was 2.2 (95% CI, 1.4 to 3.9) [4]. Sanche et al. combined case studies with model calculations and estimated that the incubation period is 4.2 days (95% CI 3.5-5.1 days) and the growth rate *r* is 0.29/day (95% CI 0.21-0.37/day), the estimated number of infected persons was ≈ 18 700 (95% CI 7 147-38 663) on January 23, 2020 [5]. Pan et al. [6] performed a cohort study using 32 583 laboratory-confirmed COVID-19 cases reported between December 8, 2019, and March 8, 2020 to determine the efficiency of public health interventions to control the COVID-19 outbreak in Wuhan [6].

In this paper, we use statistics of confirmed cases in China excluding Hubei and some other Chinese provinces as well as statistics from Beijing and Shanghai. Also, we use daily data generated by travel activity within China to analyze the daily number of infected persons in Wuhan City and Hubei Province from December 31, 2019 to January 23, 2020. In order to pursue, we develop a system dynamics (see, e.g., [7]) model that takes the infection and spread dynamics of the virus into account as well as the impact of protection measures against the epidemic such as quarantine. We employ multivariate Monte Carlo simulation (see, e.g., [8]) to parameterize the model using official statistics. In particular, we use the model to calculate the number of infections which already broke out but were not diagnosed due to various reasons.

## Methods

### Data sources

In addition to the official sources (see, e.g., [9]), we also use data from sina.com [10]. That gives daily data about cumulative, confirmed, deceased and cured cases. Crucial for this study is the data about daily travel activities on January 2020 from different parts of China, especially from the city of Wuhan and from the province of Hubei, but also from some important traffic hubs such as from Beijing. In fact, Baidu.com makes this information publicly available (see [11].

### A system dynamics model

System dynamics is an approach to understand the nonlinear behavior of complex systems over time using stocks, flows, feedback loops and non-linear functions, see, e.g., [7]. Based on the well-known SEIR model [12], we here develop a SEMIR model. This model takes the fact into account that in an epidemic situation there exist numerous infected people whose disease broke out but cannot be confirmed for various reasons. We put the emphasis on the city of Wuhan (*W*), the province Hubei excluding the city Wuhan (*H*) and China excluding the province Hubei (*C*) as three interconnected regions.

**Fig 1:**
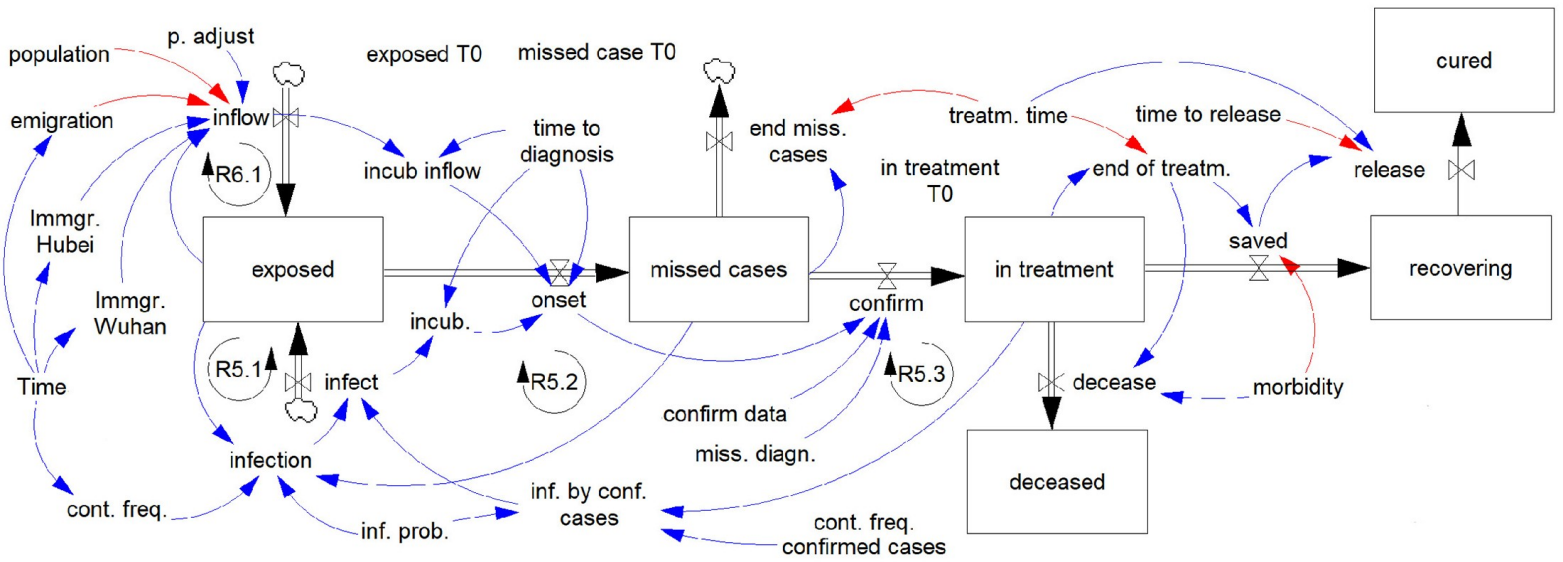
A system dynamics model for infection and spread dynamics of the SARS-CoV-2 virus.

Our system dynamics model as shown in Fig 1 is a compact visualization of an integral equation system. Each node of the graph corresponds to an equation. For each of the so-called stocks, represented by a rectangle, an integral equation applies.

Now we will explain the model step by step. An infection starts with an exposure. The exposure may take place locally or the infected persons can enter the region under consideration. The number of exposed cases is given by

$$\begin{array}{c} E_{i}\left(t\right)=E_{i}\left(0\right)+\int_{0}^{t}(f_{i}\left(\tau \right)+i_{i}\left(\tau \right)-o_{i}\left(\tau \right))d\tau \end{array}$$ (1)

where

$$\begin{array}{c} i=\{W,H,C\}. \end{array}$$ (2)

*W* stands for Wuhan, *H* for Hubei excluding Wuhan and *C* for China excluding Hubei, as mentioned above. The inflow of exposed cases is given by

$$\begin{array}{c} f_{i}\left(t\right)=f^{conv}[m_{i}^{W}\left(t\right)\frac{E_{W}\left(t\right)}{P_{W}}+m_{i}^{H}\left(t\right)\frac{E_{H}\left(t\right)}{P_{H}}-m_{i}\left(t\right)\frac{E_{i}\left(t\right)}{P_{i}}], \end{array}$$ (3)

where $m_{i}^{W}\left(t\right)$ and $m_{i}^{H}\left(t\right)$ are the daily immigrations into the region under consideration coming from Wuhan und Hubei, respectively. *m*_*i*_(*t*) is the daily exmigration from the region under consideration, *P*_*i*_ the population of the region *i*. *f*^*conv*^ is the converting factor from the points to the number of persons.

The rate of local exposure is given by

$$\begin{array}{c} i_{i}\left(t\right)=i_{i}^{E}\left(t\right)+i_{i}^{T}\left(t\right)=p[q_{i}\left(t\right)(E_{i}\left(t\right)+M_{i}\left(t\right))+q_{i}^{T}T_{i}\left(t\right)] \end{array}$$ (4)

where $i_{i}^{E}\left(t\right)$ and $i_{i}^{T}\left(t\right)$ are the exposures caused by exposed cases, *E*_*i*_(*t*), missed cases *M*_*i*_(*t*), and the ones in treatment denoted by *T*_*i*_(*t*). *p* is the probability of transmission per personal contact and *q*_*i*_(*t*) and $q_{i}^{T}\left(t\right)$ denote the contact frequency of such cases. In order to simplify, we introduce the standard contact here, where the transmission probability *p* equals 1%. Note that all non-pharmaceutical interventions, e.g., quarantine, aim to lower the frequency *q*_*i*_(*t*).

After a certain presymptomatic or incubation period *t*^*E*^, an exposed case becomes infected and will be diagnosed:

$$\begin{array}{c} o_{i}\left(t\right)=\text{delay}3\left(f_{i}\left(t-\lambda \mu t^{E}\right)+i_{i}\left(t-\lambda t^{E}\right),\left(1-\lambda \right)t^{E}\right) \end{array}$$ (5)

where the function delay3 and the parameters λ are used to approximately depict the statisitical distribution of the incubation time of the exposed cases aorund the average value *t*^*E*^ [13]

Now we analyze the so-called missed cases. If a new epidemic breaks out, it’s likely that a certain amount of infected cases are not diagosed and registered due to a lack of experience, medical capacity, or other reasons. Our model takes such possible missed cases into account:

$$\begin{array}{c} M_{i}\left(t\right)=M_{i}\left(0\right)+\int_{0}^{t}(o_{i}\left(\tau \right)-l_{i}\left(\tau \right)-c_{i}\left(\tau \right))d\tau \end{array}$$ (6)

where

$$\begin{array}{c} c_{i}\left(t\right)=\{\begin{array}{cc} o_{i}\left(t\right) & \text{if}\;m^{C}=0\\ c_{i}^{data}\left(t\right) & \text{else} \end{array} \end{array}$$ (7)

In case of

$$\begin{array}{c} m^{C}=0, \end{array}$$ (8)

all infected cases are diagnosed and registered. Otherwise only some of the cases given by the data of new cases, $c_{i}^{data}(t)$, are found and some other cases end without being diagnosed and registered. That means:

$$\begin{array}{c} l_{i}\left(t\right)=\frac{M_{i}\left(t\right)}{t^{T}}, \end{array}$$ (9)

where *t*^*T*^ is the average duration of the critical phase in which a patient may die.

The diagnosed cases are treated medically or at least observed. The following applies for the cases during treatment:

$$\begin{array}{c} T_{i}\left(t\right)=T_{i}\left(0\right)+\int_{0}^{t}(c_{i}\left(\tau \right)-d_{i}\left(\tau \right)-s_{i}\left(\tau \right))d\tau . \end{array}$$ (10)

The following applies to deceased and rescued cases:

$$\begin{array}{c} d_{i}\left(t\right)=r_{i}^{D}\frac{T_{i}\left(t\right)}{t^{T}}, \end{array}$$ (11)

$$\begin{array}{c} s_{i}\left(t\right)=\left(1-r_{i}^{D}\right)\frac{T_{i}\left(t\right)}{t^{T}}, \end{array}$$ (12)

where $r_{i}^{D}$ is the mortality of the disease under consideration.

Finally we discuss the features of the model when analyzing cases of death, recovered and cured cases. To be able using the official statistics for our calculation, we consider the cases of death and cured cases as well as the recovered cases but have not yet been released from medical treatment:

$$\begin{array}{c} D_{i}\left(t\right)=D_{i}\left(0\right)+\int_{0}^{t}d_{i}\left(\tau \right)d\tau , \end{array}$$ (13)

$$\begin{array}{c} C_{i}\left(t\right)=C_{i}\left(0\right)+\int_{0}^{t}r_{i}\left(\tau \right)d\tau , \end{array}$$ (14)

$$\begin{array}{c} R_{i}\left(t\right)=R_{i}\left(0\right)+\int_{0}^{t}(s_{i}\left(\tau \right)-r_{i}\left(\tau \right))d\tau , \end{array}$$ (15)

where

$$\begin{array}{c} r_{i}\left(t\right)=s_{i}\left(t-t^{R}+t^{T}\right). \end{array}$$ (16)

*t*^*R*^ is the total time from the diagnosis of a case to its full recovery.

### Parameterization

The model shown in Fig 1 and in the previous section contains a number of parameters. In the first step we neglect the exposure due to diagnosed cases, i.e. set $q_{i}^{T}\left(t\right)=0$, to focus on those parameters such that the model can reflect the actual infection and spread of COVID-19. These parameters include:

- *E*_*W*_(0) is the number of exposed cases in Wuhan on Dec. 31th 2019
- *f*^*conv*^ is the converting factor from the points to number of persons
- *q*_*W*_(0) and *q*_*H*_(0) are the contact frequencies in Wuhan and Hubei before Jan. 23rd 2020
- *t*^*E*^ is the time between the exposure and the diagnosis
- λ is used to describe the the statisitical distribution of *t*^*E*^
- *μ* is used to descibe the proportion of *t*^*E*^ which is spent in the target region
- *q*_*C*_(*t*) is the time function of contact frequency in China excluding Hubei *C* which is given by four keypoints representing the contact frequency on Jan. 21st, Jan 25th, Feb. 10th and Feb. 27th, 2020.

**Fig 2:**
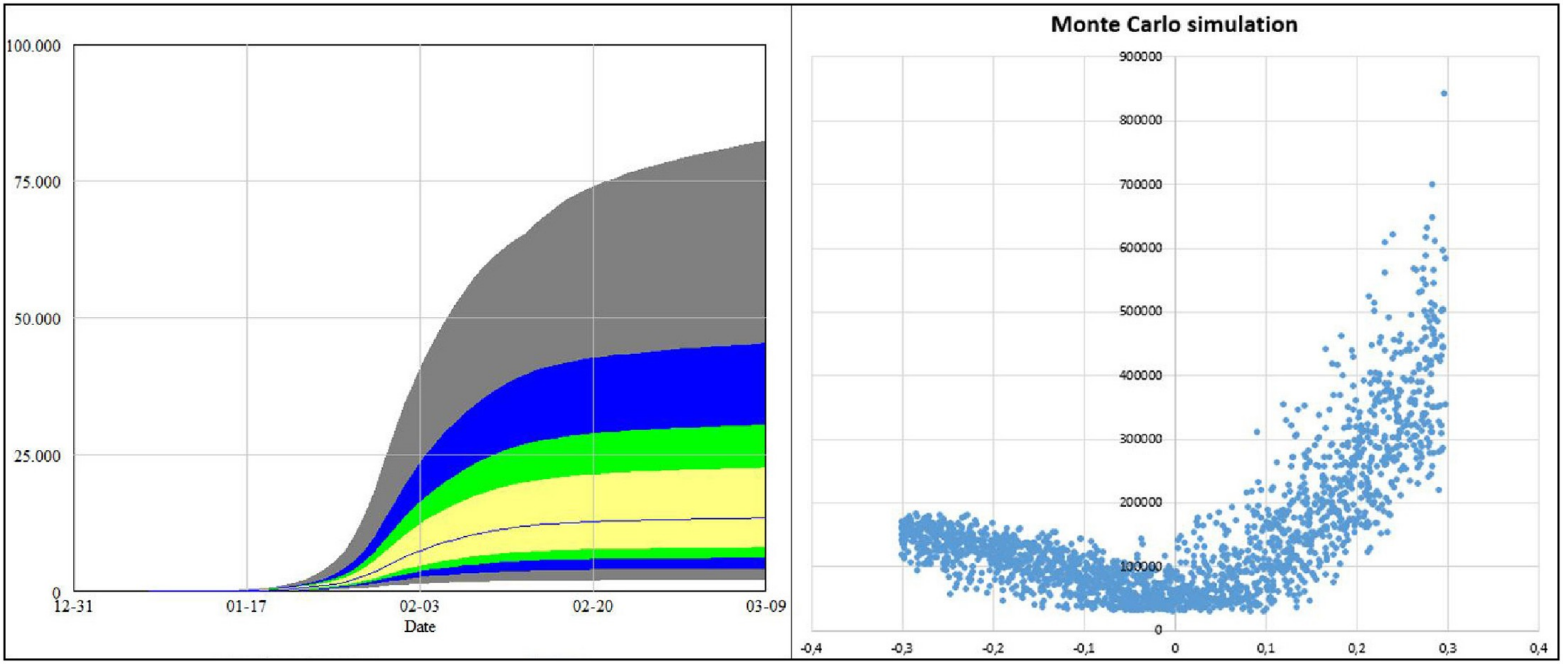
Monte Carlo simulations for finding the parametrization.

By using multivariate Monte Carlo optimization, a large number (e.g., 10 000) of simulation runs are carried out with randomly generated values for the above mentioned parameters. The sensitivity results from these simulation runs are compared with the real data. (Fig 2). Hence, we obtain the appropriate values thereof and also confidence intervals for these parameters. However, since the daily departures from Wuhan and Hubei are considered by using random samples of the local population, the values for the confidence interval of the parameters that apply to Wuhan and Hubei increase accordingly. To keep the confidence interval within 15%, we focus on the period of time between January 17th through 26th where both the travel activity and the proportion of infected cases were at a relatively high level. Incidentally, a repetition of the simulation runs with other reasonable values of $q_{i}^{T}$ shows that the changes in the results compared to the confidence interval specified here are negligible.

## Findings and interpretations

Using the method described in Section ‘Methods’, we find that the infection rate before the quarantine in Wuhan was 22.3% ± 3.3% (95% confidence interval, CI) per day. We determine the average time between exposure and confirmed diagnosis by 10.2 ± 1.5 days (95% CI). This yields to the reproduction number R equal to 2.27 ± 0.34 (95% CI) before the quarantine in Wuhan was arranged. Also, we estimate that the number of exposed cases based on the SEMIR model was 49 800 ± 7500 (95% CI) on January 23rd, 2020; this is the day the quarantine was arranged in Wuhan. More than 5 000 of these cases were active, although only 495 cases had been registered by that time. The number of infected cases which were not diagnosed for various reasons peaked at 28 200 ± 4 200 on February 04th 2020.

**Fig 3:**
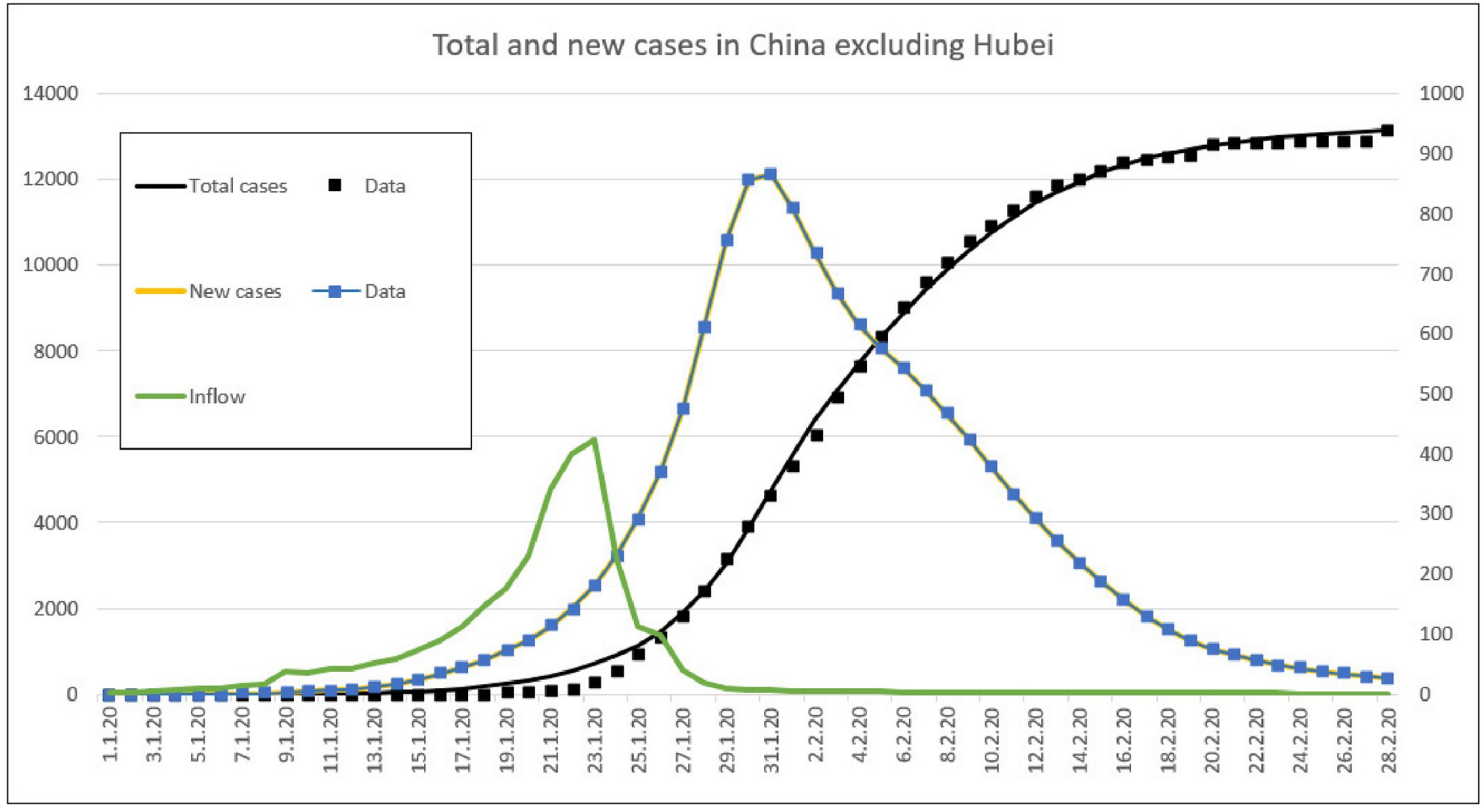
Statistical data and simulated course of the pandemic COVID-19 in China excluding Hubei.

As shown by Fig 3, the model simulation can reproduce the actual course of the pandemic COVID-19 in China apart from Hubei. In addition, it provides information on the daily numbers of cases flowed in from Wuhan and Hubei and locally exposed cases. It is remarkable that non-pharmaceutical interventions actually work out in order to reduce the country-wide infection rate from 19% per day to ≤ 3% (or contact frequency from 19 per day to 3, a reduction of more than 80%) so that the spread of the pandemic was largely stopped within China.

**Fig 4:**
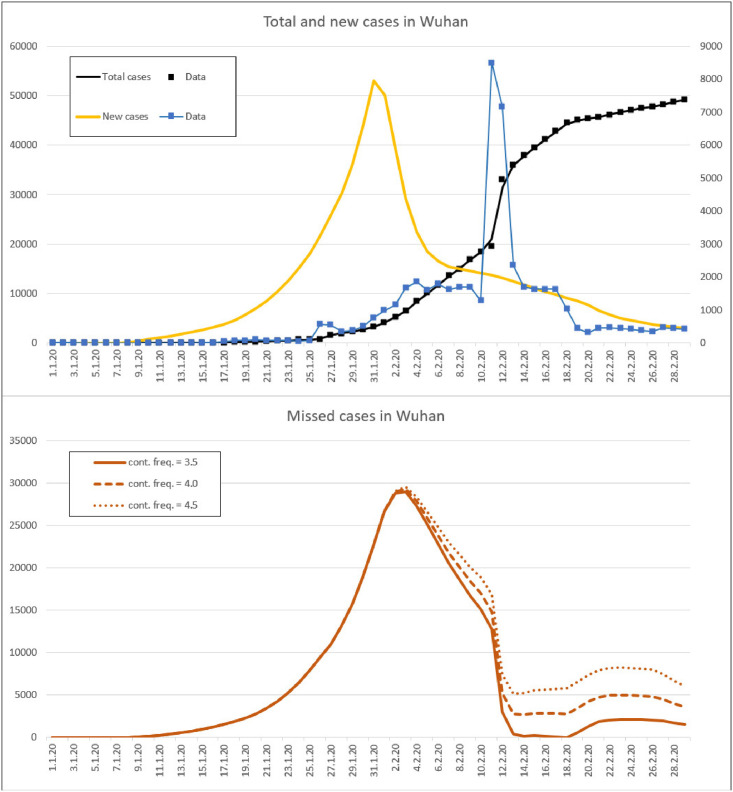
Statistical data and simulated course of the pandemic COVID-19 in Wuhan.

The situation in Wuhan until shortly after quarantine can also be determined with the present method. On February 3, there were almost 30 000 missed cases. However, we cannot determine how Wuhan then developed in this regard with the actual method. Fig 4 shows three different scenarios in which the contact frequency is 3.5, 4.0 and 4.5 per day, respectively.

## Conclusions

In this paper, we came up with an estimate of the number of infected people in Wuhan to obtain a consistent, quantitative picture of the overall situation of a pandemic for connected areas. With our System Dynamics model, we took into account not only the infection dynamics, but also the spread through the travel activities and the anti-epidemic measures conducted. Our study revealed that the model was able to capture significant information to analyze the real number of infected people in Wuhan. Compared to foregoing studies discussed in the Section ‘Introduction’, our estimates are more accurate and the system dynamics model incorporates relevant factors such as quarantines imposed by the Chinese government.

Based on the model introduced in this article, we are closely monitoring the development of the COVID-19 pandemic in various countries and regions, observing the effect of different isolation methods and detection strategies in real time. Through data analysis, parameters such as infection rate, test coverage and immunity rate are obtained. Our aim is to contribute to contain the pandemic.
